# Synchronous clear cell renal cell carcinoma and tubulocystic carcinoma: genetic evidence of independent ontogenesis and implications of chromosomal imbalances in tumor progression

**DOI:** 10.1186/1746-1596-7-21

**Published:** 2012-02-27

**Authors:** Gabriela Quiroga-Garza, Sergio Piña-Oviedo, Karime Cuevas-Ocampo, Richard Goldfarb, Mary R Schwartz, Alberto G Ayala, Federico A Monzon

**Affiliations:** 1 Department of Pathology and Genomic Medicine, The Methodist Hospital, Houston, Texas 77030, USA; 2 Department of Pathology and Laboratory Medicine, Weill Cornell Medical College, New York, New York, USA; 3 Departamento de Patología, Centro Médico Nacional Siglo XXI, México D.F. 06720, Mexico; 4 Department of Urology, The Methodist Hospital, Houston, TX 77030, USA; 5 Department of Pathology and Genomic Medicine, The Methodist Hospital, Houston, TX 77030, USA; 6 Cancer Genetics Laboratory, Baylor College of Medicine, Houston, TX 77021, USA

**Keywords:** Renal cell carcinoma, Collecting duct carcinoma, Tubulocystic carcinoma, Cystic tubules, Genetic profile, Virtual karyotyping, Tumor progression, Fuhrman nuclear grade, Synchronous tumor

## Abstract

Seven percent of renal cell carcinoma (RCC) cases are diagnosed as "unclassified" RCC by morphology. Genetic profiling of RCCs helps define renal tumor subtypes, especially in cases where morphologic diagnosis is inconclusive. This report describes a patient with synchronous clear cell RCC (ccRCC) and a tubulocystic renal carcinoma (TCRC) in the same kidney, and discusses the pathologic features and genetic profile of both tumors. A 67 year-old male underwent CT scans for an unrelated medical event. Two incidental renal lesions were found and ultimately removed by radical nephrectomy. The smaller lesion had multiple small cystic spaces lined by hobnail cells with high nuclear grade separated by fibrous stroma. This morphology and the expression of proximal (CD10, AMACR) and distal tubule cell (CK19) markers by immunohistochemistry supported the diagnosis of TCRC. The larger lesion was a typical ccRCC, with Fuhrman's nuclear grade 3 and confined to the kidney. Molecular characterization of both neoplasms using virtual karyotyping was performed to assess relatedness of these tumors. Low grade areas (Fuhrman grade 2) of the ccRCC showed loss of 3p and gains in chromosomes 5 and 7, whereas oncocytic areas displayed additional gain of 2p and loss of 10q; the high grade areas (Fuhrman grade 3) showed several additional imbalances. In contrast, the TCRC demonstrated a distinct profile with gains of chromosomes 8 and 17 and loss of 9. In conclusion, ccRCC and TCRC show distinct genomic copy number profiles and chromosomal imbalances in TCRC might be implicated in the pathogenesis of this tumor. Second, the presence of a ccRCC with varying degrees of differentiation exemplifies the sequence of chromosomal imbalances acquired during tumor progression.

**Virtual Slides:**

The virtual slide(s) for this article can be found here:

http://www.diagnosticpathology.diagnomx.eu/vs/1790525735655283

## Introduction

Kidney cancer is among the ten most common causes of cancer-related death in adults [1]. Over 64,700 new cases and over 13,500 deaths are expected to occur in the US in 2012 [2]. Renal cell carcinoma (RCC) constitutes more than 80% of all primary renal neoplasms, and clear cell RCC (ccRCC) accounts for most of these cases (80%). While a specific histological diagnosis is possible by morphology and immunohistochemistry in the majority of cases, overlapping morphological features are still encountered in about 7% of cases in routine practice [3], and these cases fall in the category of "unclassified renal neoplasm." Recent updates to the histopathological diagnosis of kidney neoplasms are reflected in the latest World Health Organization (WHO) classification of genitourinary and kidney neoplasms [4]. However, ongoing research has led to the description of new tumor entities that are not yet considered in this classification. One of these recently described entities is tubulocystic renal carcinoma (TCRC) with less than one hundred cases reported [5].

Genetic events play an important role in renal carcinogenesis, and most known renal tumor subtypes have recurrent specific chromosomal imbalances [6]. Thus, genetic profiling of renal tumors has emerged as a practical diagnostic tool for the surgical pathologist, especially in those cases where morphologic diagnosis is not conclusive [3]. Although the identification of key molecular targets involved in renal carcinogenesis has had direct impact in patient care, little is known about the genetic events involved in tumor progression [6].

Herein we describe the clinico-pathological features of a patient presenting with synchronous ccRCC and a TCRC and the molecular profiles of these tumors utilizing virtual karyotyping. Our results contrast the different profiles of these tumors as well as exemplify the chromosomal imbalances associated with histologic progression from low to highgrade ccRCC.

## Case presentation

**Figure 1:**
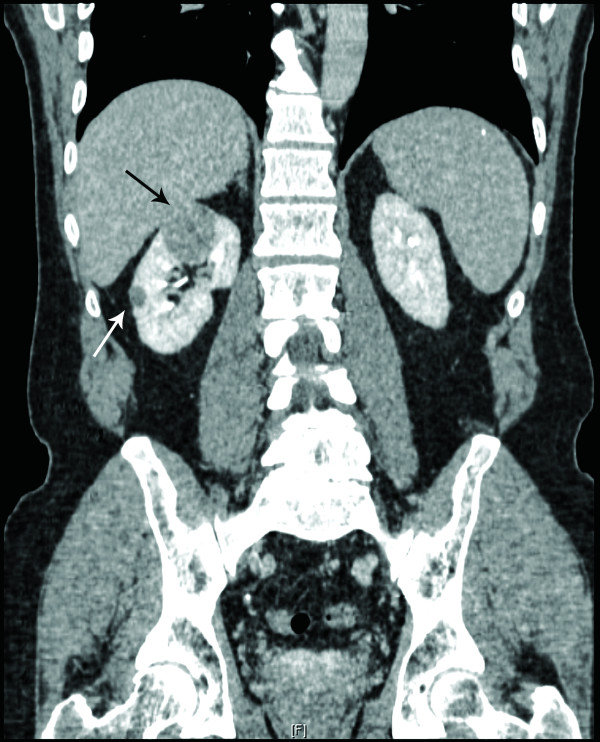
**Imaging, Computed tomography (CT) showed the presence of two incidental well-circumscribed masses with similar density in the right kidney**. The larger lesion was solid and enhancing and measured 3.5 cm; it was located in the upper pole and approached but did not extend into the renal sinus (black arrow). The smaller lesion measured 1.2 cm and was located along the mid lateral aspect of the kidney (white arrow). The contralateral kidney was normal.

A 67-year-old previously healthy male presented with acute onset of chest pain. His past medical history was unremarkable except for a history of RCC in his mother. The patient's physical exam and cardiac workup were negative. Two incidental right renal masses were found on CT scan performed as part of his chest pain evaluation (Figure 1). A 3.5 cm, solid, enhancing mass located in the anterior aspect of the upper pole; and a 1.2 cm mass located along the mid lateral aspect of the kidney. The patient denied flank pain, hematuria, or weight loss. Additional workup for metastatic disease was negative. Partial nephrectomy was attempted to remove the lesions; however, a total nephrectomy was ultimately necessary. No adjuvant therapy was administered to the patient. Twenty-four months after the procedure, the patient remains free of disease.

### Gross and microscopic findings

**Figure 2:**
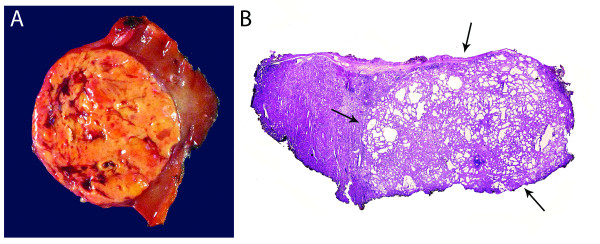
**Gross image and full-montage of the ccRCC and TCRC, respectively. A) The larger mass was well encapsulated, tan-yellow to red and grossly suggestive of ccRCC**. This lesion was not associated with the smaller nodule. B) Full-montage of the smaller lesion. The 'spongy' nodule encountered in the gross description (arrows) was composed of cystically-dilated irregular tubules of different sizes, separated by fibrovascular septae (H&E).

At surgery, a wedge biopsy including the smaller lesion was initially submitted for frozen section and was interpreted as TCRC vs. cystic nephroma, followed by a partial nephrectomy of a larger lesion interpreted as a ccRCC. The smaller nodule (2 × 1.7 × 1 cm) was a tan-gray small sphere with a spongy cut surface that was adjacent to a 0.3 cm hemorrhagic cyst. The larger tumor was a relatively well encapsulated tan-yellow to red mass (4.1 × 3.4 × 2.5 cm) macroscopically compatible with ccRCC which showed no association with the previous smaller lesion (Figure 2A). A radical nephrectomy was ultimately performed.

**Figure 3:**
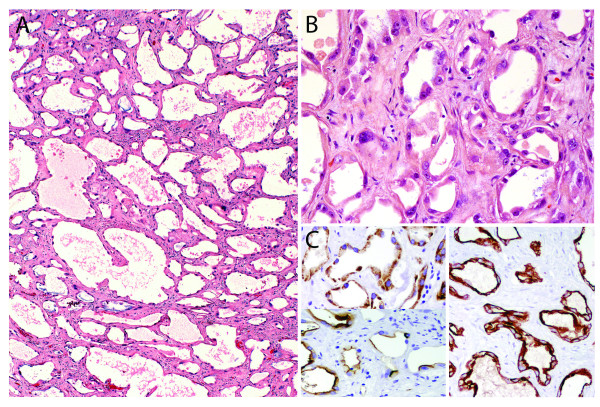
**Tubulocystic carcinoma component**. **A**) Higher magnification of the image shown on figure 2B (H&E, 4×). **B**) The cystically-dilated tubules were lined by flat to polygonal cells (some with a hobnail profile) with enlarged nucleus, prominent nucleolus, and occasional abundant eosinophilic cytoplasm. The fibrous septae contained scattered fibroblast-like spindle cells interspersed within a collagenous stroma (H&E, 20×). C) Tubular cells were positive for AMACR (left, top) and CD10 (left, bottom), and CK19 (right) confirming their proximal and distal convoluted tubule cell origin, respectively (all images, 20×). Estrogen receptor was negative in both epithelial and stromal elements (not shown).

**Figure 4:**
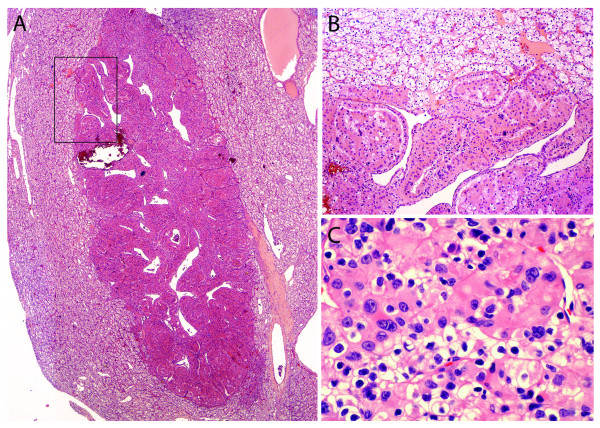
**Clear cell RCC component**. **A**) The largest lesion described in the gross description was composed of cells with abundant clear cytoplasm and well-demarcated cellular borders. Marked oncocytic changes with a nodular configuration were present in some areas (H&E, 2×). **B**) The rectangle in A at higher magnification shows the transition between the clear (top) and oncocytic cells (bottom). Nuclear features in the clear cell areas were consistent with Furhman nuclear grade 2, whereas the oncocytic nodule's cells showed increased pleomorphism and slightly enlarged nucleoli (H&E, 10×). **C**) A separate area of the tumor showed clusters of tumor cells with marked nuclear pleomorphism, irregular nuclear contours, and prominent nucleoli, consistent with Furhman nuclear grade 3 (H&E, 40×).

Microscopically, the smaller lesion was composed of multiple cystic tubules of variable size separated by fibrovascular septae (Figure 2B, arrows). The tubules were lined by flat to polygonal and epithelioid cells displaying a prominent hobnail pattern. There was nuclear enlargement, prominent nucleoli, and eosinophilic cytoplasm (Figure 3A-B), but mitotic figures were not observed. Some tubules occasionally contained intraluminal clumps of eosinophilic acellular material. By immunohistochemistry, the tubular cells were positive for alpha-methylacyl-CoA racemase (AMACR), CD10 and cytokeratin 19 (CK 19), which are markers commonly positive in TCRC tumors (Figure 3C) [7,8], while the stromal cells were negative for estrogen receptor. In contrast, the larger tumor was primarily composed of nests of cells with abundant clear cytoplasm with well-demarcated cellular borders and hyperchromatic nuclei with inconspicuous nucleoli (Furhman nuclear grade 2) separated by a well-formed capillary network. In some areas, the tumor showed well-demarcated nodules with oncocytic change together with mild nuclear pleomorphism and slightly enlarged nucleoli (Figure 4A-B). Additionally, a focal area of the tumor contained cells with marked pleomorphism, irregular nuclear contours, and prominent nucleoli (Furhman nuclear grade 3) (Figure 4C). Sarcomatoid features were not present. A final diagnosis of synchronous TCRC and ccRCC, Fuhrman nuclear grade 3, Stage T1b, was established.

### Virtual karyotyping

**Figure 5:**
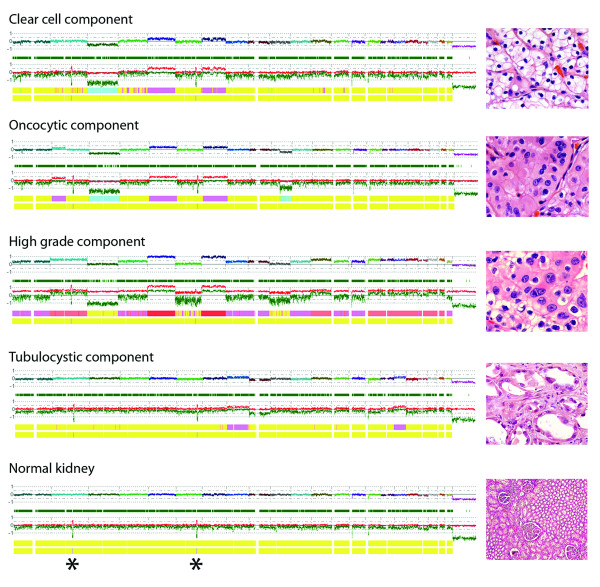
**Genomic profile of tumor components**. The uppermost plot for each sample represents the estimated copy number as a log 2 ratio averaged over 30 SNPs; middle bar represents a color-coded Hidden Markov Model (HMM) for copy number (yellow = copy number 2, pink = copy number 3, aqua = copy number 1), and bottom bar is a color-coded HMM for LOH (yellow = no LOH, blue = LOH). Genomic analysis revealed loss of chromosome 3p and gains in chromosomes 5 and 7 in all ccRCC components, a gain of 2p and loss of 10q in the oncocytic component, and a profile consistent with polyploidy (likely trisomic) with additional gains and losses in the high-grade component. The tubulocystic component showed a profile distinct from that of ccRCC. Germline uniparental disomy (UPD) of chromosomes 2 and 6 [UPD2 (q21.3-q22.1), UPD6 (q23.2-q23.3)] (asterisks) was found in all (tumor and normal) specimens from the patient.

DNA was extracted from microdissected tumor tissue from the clear cell and oncocytic areas with Fuhrman grade 2, the region of ccRCC with Fuhrman grade 3, from the TCRC, and from uninvolved kidney tissue. Virtual karyotyping was done using Affymetrix 250 K *Nsp *SNP (single nucleotide polymorphism) arrays as described previously [3]. Results for SNP oligonucleotide microarray karyotype analysis for each region of the tumors and normal kidney are shown in Figure 5. All analyzed areas of the ccRCC showed characteristic loss of chromosome 3p and gains in chromosomes 5 and 7. In addition to these findings, the area of oncocytic change showed a gain of 2p and a loss of 10q. The area of tumor with Fuhrman nuclear grade 3 exhibited a profile consistent with polyploidy (likely trisomic) with the following additional imbalances: +2,+5,-6,+7,+10,+12,+15,+16,+17,+18,+19,+20,+21. The TCRC displayed a profile distinct from that of the ccRCC, with gains of chromosomes 8 and 17 and loss of chromosome 9. The non-neoplastic kidney showed no chromosomal gains or losses. However, uniparental disomy in regions of chromosomes 2 and 6 [UPD2 (q21.3-q22.1), UPD6 (q23.2-q23.3)] was found in tumoral and non-neoplastic kidney from the patient (asterisks, Figure 5), indicating a germline abnormality.

## Discussion

We report a case of two synchronous non-related tumors, a TCRC and a ccRCC occurring in the same kidney with molecular characterization of both neoplasms.

TCRC was originally described by Masson who named it "Bellinian epithelioma" or "carcinoma of the collecting ducts" as he believed it originated from collecting ducts of Bellini [9]. MacLennan et al. [10] in 1997 hypothesized that this tumor represented the low grade of the spectrum of collecting duct carcinoma, as it shares similar characteristics with the latter tumor (i.e., immunopositivity for 34βE12 and UEA-1). In 2004, Amin et al. named the tumor "tubulocystic carcinoma" in a series of 29 cases [11].

TCRC is a rare entity that characteristically has a small size at presentation (mean 2 cm) and rarely progresses, recurs, or metastasizes [7,8,12,13]. It has been reported in association with other RCCs, primarily papillary RCC [5]. Recent immunohistochemical, ultrastuctural, and molecular studies (RNA expression levels and genomic profile analyses) have shown that TCRC is different from collecting duct carcinoma and may be related to papillary RCCs [5,7,8,13-15]. Supportive findings include the expression of proximal convoluted tubule markers (CD10 and AMACR), distal nephron proteins (parvalbumin, HMWCK and CK19) [5,7], and the detection of cells reminiscent of proximal tubule and intercalated cells by electron microscopy [5,13-15]. Further supporting the notion that TCRC is related to papillary RCC is the report of one group that described that TCRC has a gene expression profile similar to that of papillary RCCs using cluster analysis [8]. However, another study showed a distinct gene expression profile characterized by expression of cell cycle and biomolecule metabolism genes although this group did not compare this profile directly to papillary tumors [5]. Using fluorescence in situ hybridization (FISH) and array-based comparative genomic hybridization (aCGH), Zhou et al. [13] and Yang et al. [8] have shown gains in chromosome 17, as is seen with papillary RCCs and thus have proposed that these two entities are closely related.

Our case showed classic features of TCRC, such as positive immunohistochemistry for CD10, AMACR and CK19, as well as gains of chromosome 17 in the virtual karyotyping analysis. In addition, the TCRC also showed gains in chromosome 8 and loss of chromosome 9 (Figure 5). The chromosomal imbalance pattern observed in our TCRC case is distinct from that found in papillary RCC which commonly shows gains of 7 and 17 and rarely shows gains of 8 or loss of 9 [6]. Importantly, loss of chromosome 9 and gain of 17 seem to be recurrent chromosomal imbalances in TCRC tumors (Monzon and Amin, unpublished observations). If this is confirmed in a large case series, it could help establish TCRC as a genetically separate entity from papillary RCC. It is important to note that the cystic and somewhat paucicellular nature of the tumor may be an important consideration when performing molecular analyses of this tumor subtype, since stromal cells may play a role as contaminants. Also, the role of stromal cells in the pathogenesis of TCRC remains uncertain, since metastatic cases not only show the cystic glandular configurations but also the intervening stroma [5].

The differential diagnosis of TCRC includes mixed epithelial and stromal tumor and cystic nephroma [16]. The former, another variant of RCC, is easily ruled out as typically displays an ovarian-like stroma that is usually estrogen receptor positive and occurs predominantly in female patients [17,18]. The so-called cystic nephroma has become basically a non-existent entity [17,19]. In the WHO classification of renal tumors [4] there is an illustration of a cystic nephroma which looks identical to the TCRC. We suspect that the "cystic nephroma" is actually the tumor now known as TCRC.

There are sporadic reports about the coexistence of TCRC with other renal tumors of different subtypes. Yang et al. reported 5 cases of TCRC associated with either papillary RCC or papillary adenomas [8]. Gonul et al. reported a case of synchronous ccRCC, micropapillary urothelial carcinoma and TCRC in a 57-year-old male with hematuria [20]. Brennan et al. presented a case of a 72-year-old male with end stage renal disease who developed a TCRC, a type 2 papillary RCC, a clear cell papillary and cystic RCC as well as renal oncocytosis, hybrid tumors and chromophobe RCC [21]. More recently, Deshmukh et al. reported a synchronous TCRC and papillary RCC in a young female with metastatic papillary RCC in para-aortic lymph nodes [22]. Thus, although TCRC has been reported in association with multiple other renal cell tumor subtypes, it appears that there is a slight predominance for synchronous TCRC and papillary tumors. An interesting question raised by the coexistence of TCRC with other renal tumors is whether there are common predisposing factors for these histologically different tumors.

The ccRCC in this case showed a spectrum of morphologic variations ranging from low grade to high grade and a distinct nodule with oncocytic changes which allowed us to inquire about molecular differences within morphologic progression in a single tumor. We analyzed the areas in the ccRCC showing different grade and morphology using virtual karyotyping and demonstrated that a higher Fuhrman grade correlated with an increase in chromosomal imbalances (Figure 5). In a similar fashion, studies on chromophobe RCCs have shown that multiple chromosomal gains are associated with sarcomatoid transformation [23]. More recently, Patani et al. using high-resolution aCGH and FISH have also demonstrated that morphologically distinct regions from a case of triple-negative breast carcinoma (apocrine vs. non-apocrine areas) correlate with different genetic aberrations [24].

In addition to the somatically acquired genetic changes in the tumors, the patient's tumor had regions of inherited uniparental disomy (UPD, i.e. two chromosomal regions inherited from one parent only) in chromosomes 2 and 6 (Figure 5, asterisks). The presence of these genetic lesions in the normal kidney tissue suggests a possible inherited origin. Although uniparental disomy has been implicated as a risk factor for colon and other cancers, its role in the predisposition of renal tumors is currently unknown [25].

In conclusion, this case allowed us to explore two uses of whole genome analysis with virtual karyotyping. The first was to confirm that ccRCC and TCRC have distinct genomic copy number profiles and that TCRC appears to have recurrent gains in chromosome 17 which might be implicated in the pathogenesis of this tumor. Second, the presence of a ccRCC with varying degrees of differentiation allowed us to evaluate the sequence of chromosomal imbalances acquired during tumor progression. These findings support the concept that chromosomal imbalances are associated with tumor progression [26], and possibly explain why Fuhrman grading is a strong prognostic factor in ccRCC [27] even when high grade cells are present in a minority of the specimen [28].

## Consent

This study was conducted under a local IRB approved protocol for the molecular characterization of renal tumors.

## Competing interests

The authors declare that they have no competing interests.

## Authors' contributions

GQG and SPO retrieved clinical information and wrote the manuscript. RG, GQG, KCO, MRS and AA first identified this case, proposed the studies and provided valuable insight during manuscript preparation. FAM provided the molecular analysis data and supervised manuscript preparation. All authors reviewed and approved the final manuscript.
